# Hypoglycemia in the hospital

**DOI:** 10.3402/jchimp.v1i2.7217

**Published:** 2011-07-18

**Authors:** Mansur Shomali

**Affiliations:** Department of Medicine, Union Memorial Hospital, Baltimore, MD, USA

**Keywords:** hypoglycemia, hospital, diabetes, insulin

## Abstract

Hypoglycemia is a common adverse event affecting hospitalized patients with diabetes. This paper reviews the data regarding optimization of glucose in hospitalized patients, discusses the scope and significance of hypoglycemia in the hospital, and makes recommendations on how to reduce the risk of this serious adverse event.

As an internal medicine faculty member at a community teaching hospital, I inherited a medical team recently and inquired about a patient, Mrs. K, whom I noticed had been in the hospital almost a month. The residents calmly recited the facts of the case: she was a 71-year-old woman with known coronary disease, a history of congestive heart failure, chronic kidney disease, and type 2 diabetes mellitus who was admitted with an infected foot ulcer but was getting ‘better’. She had recovered from septicemia and had a below-knee amputation. Hardly mentioned was the fact that the previous night, her blood sugar was found to be 43 mg/dL. Her anti-diabetic medications were insulin glargine 20 units and ‘sliding scale’. This case raises some interesting questions. What target glucose should we, the patient's medical team, aim for? What insulins should we use and how should their doses be adjusted? What were the potential causes of the hypoglycemia? Could it have been avoided?

With the publication of studies on DM1 (1) and DM2, (2) keeping the blood sugar closer to normal to prevent long-term complications became the standard of care. Unfortunately, better control of these outpatients resulted in more hypoglycemia. Because the benefits of better glucose control were seen in patients over several years, the care of these patients during brief hospitalizations did not change very much. The major strategy was permissive hyperglycemia to avoid the acute danger to the hospitalized patient: hypoglycemia. This strategy changed about 10 years ago with the publication of the pivotal study by van den Berghe et al. (3) which showed a major reduction in mortality and other serious complications in critically ill surgical patients who were treated to a target blood sugar of between 80 and 110 mg/dL. Influential consensus statements were published by professional societies broadly recommending these new targets (4). Many hospitals, mine included, created glycemic control teams and protocols to address the hyperglycemia problem. Fueling our efforts were numerous in vitro studies linking hyperglycemia to oxidative stress, inflammation, immune dysfunction, and endothelial cell abnormalities (5). These studies teased us with biological plausibility.

A number of studies have been published since then that have dampened the exuberance for the 80 to 110 mg/dL glucose targets, the most important of which was the NICE-SUGAR study in critically ill patients (6). In fact, the group with tight glycemic control in NICE-SUGAR had higher 90-day mortality than the less-intensively treated control group. From an outpatient standpoint, the ACCORD study also showed increased cardiovascular mortality in an intensively treated population of patients with DM2 (7). Several professional organizations have since revised their guidelines and now suggest blood sugar may go up to 180 mg/dL for most hospitalized patients (8).Although it has not been clearly proven, the pervasive opinion is that the inability to improve inpatient outcomes in patients with diabetes by controlling hyperglycemia may be due to hypoglycemia. It is not that glucose control does not matter but that the risk of hypoglycemia may be negating the benefit of treating hyperglycemia in many of these studies.

Hypoglycemia is quite common. In a publication of data from the University Hospital Consortium, the prevalence of severe hypoglycemia, defined as a blood sugar value of <40 mg/dL ranged from 3 to 11% (9). According to unpublished data, the overall rate of BG < 40 mg/dL at our hospital system of seven community hospitals is approximately 3.4%, with substantial variation among hospitals.

Hypoglycemia in the hospital has been shown to have adverse consequences. In a study by Turchin et al. (10) hypoglycemia during a single hospital day increased the length of stay by 1 day, and hypoglycemia recorded on two separate days increased the length of stay by over 2 days. In the same study, exposure to hypoglycemia increased the 1-year mortality rate proportionally to the number of days that hypoglycemia was observed (9). In August 2008, the Inpatient Prospective Payment System expanded the list of hospital-acquired conditions that affect Medicare payments. These ‘never events’ took effect in October 2008 and included ‘manifestations of poor blood sugar control such as manifestations of severe hypoglycemia’ (11).

Hospitalized patients with diabetes develop hypoglycemia for a number of reasons, many of which can be recognized and avoided (Table 1). A common cause is the administration of oral medications in the sulfonylurea class, which includes the drugs glyburide, glipizide, and glimeperide. Sulfonylureas potentiate insulin secretion by the pancreas. If a hospitalized patient misses a meal or consumes fewer carbohydrates than usual, hypoglycemia may result with these medications. A simple solution to avoid sulfonylurea-associated hypoglycemia is to not prescribe these medications in the hospital, especially in very sick, unstable patients.

**Table 1:** Common causes of hospital hypoglycemia and strategies for reducing the risk

Potential cause of hypoglycemia	Potential strategies for reducing risk
Dose of mealtime insulin was administered but patient did not consume enough carbohydrates	Nurses should be trained to administer the rapid-acting mealtime insulin dose when the meal tray has arrived and patient is ready to eat; if there is a question as to whether the patient will eat the majority of food on his/her tray, the nurse should wait to administer the mealtime insulin dose.
The wrong brand of insulin or an incorrect dose was transcribed from the physician order	Hospital should only allow insulin to be prescribed using special order sets. Hospital formularies should be simplified to include one basal and one rapid acting insulin analog. The abbreviation ‘U’ for unit should be disallowed. U-500 concentrated insulin should be restricted by the pharmacy.
Patient became hypoglycemic after a dose of mixed insulin was given	In general, mixed insulin should not be used in the hospital setting. Patients using mixed-insulin outside the hospital can be converted to basal and rapid-acting insulin during the admission. The total daily dose of mixed insulin is summed. Half of this number is then given as basal insulin and the other half is given as mealtime insulin in patients who are eating. For example, if a patient is taking lispro 75/25 mix, 40 units in the morning and 20 units at night, the hospital regimen would be 30 units ([40 + 20]/2) of basal insulin once daily and 10 units of mealtime insulin with each meal.
Patient's dose of steroids was reduced	Medical providers should be trained to pre-emptively adjust insulin doses as clinical status of the patient changes.
Patient recovered from illness and stress response waned, thus reducing insulin resistance	Patients whose clinical status or severity of illness is changing should be monitored carefully. Medical providers should be encouraged to adjust insulin doses daily as needed to achieve targets (e.g., 110 to 180 mg/dL) set by the safety officer or medical director of the hospital.
Patient was treated with correctional insulin only, not basal and mealtime insulin (sliding scale)	Medical providers should be trained in the appropriate use of insulin therapy. Sliding scale as the only means of insulin therapy should be restricted or discouraged.
Systemic problems on particular units result in hypoglyemia; e.g., physical therapy during acute rehabillitation admission	Hospital performance improvement nurse or administrator should monitor hypoglycemia incidence and direct interventions to appropriate departments. Attending physicians, house staff, nurse practitioners, physician assistants, nurses, and unit secretaries should receive training in-services and updates on care of the diabetes patient.
Patient was treated with oral-hypoglycemic agents	Use of oral anti-diabetic medications should be avoided in most hospitalized patients.

Insulin therapy is the preferred method for treating hyperglycemia in the hospital. The vast majority of hypoglycemia is due to issues with insulin dosing. Simple dosing errors such as confusing the letter ‘U’ of ‘units’ with a zero, thus making ‘3 U’ appear like ‘30’ units can be avoided by banning the abbreviation and using insulin order sets. Careful nursing procedures should be implemented to avoid injecting insulin instead of another injectable medication such as heparin.

In my experience, most hypoglycemia is not due to these simple dosing errors; rather it is due to the complexity of selecting the optimal dose and type of insulin in patients whose insulin requirements are unknown and whose severity of illness and carbohydrate intake is fluctuating. Insulin analogs are preferred over the human insulins due to predictability of response. Mixed insulins should be avoided because the peak effect may occur at a time when the patient is not eating or the patient may be away from the nursing unit for a procedure, test, or therapy session.

Patients should not be prescribed correctional insulin (or ‘sliding scale’ insulin as it is commonly called) as the only insulin therapy, but rather should be given a combination of long-acting and fast-acting analogs to meet their insulin requirements. Sliding scale insulin use has been associated with severe hypoglycemia, particularly in patients who are very sensitive to insulin and, if prescribed, should be used with extreme caution (12). Correctional insulin may be used judiciously as a supplement to basal and meal-time insulin, in order to correct pre-meal hyperglycemia. When used this way, the term ‘sliding-scale’ is not used; it is referred to as ‘supplemental’ or ‘correctional’ insulin.

Long-acting insulin analogs (either glargine or detemir) should be prescribed to meet basal insulin requirements. Patients who are sick generally have increased basal needs due to the physiologic stress response (13). The basal insulin should be adjusted based on the fasting blood sugars of the patient. The dose should be titrated up if the fasting glucose is above target (for example, >140 mg/dL) or down if the fasting glucose is below target (for example, <110 mg/dL). Pre-emptively lowering the dose as a patient recovers and the stress response wanes may be prudent. Pre-emptive adjustments may also be done in patients treated with glucocorticoids. For example, if a patient is treated with prednisone for an asthma exacerbation and the steroid dose is lowered but the insulin dose is not lowered, hypoglycemia may occur the next day (see Table 2).

**Table 2:** Guide for adjusting insulin doses to avoid hypoglycemia

Event	Suggested action
Fasting blood glucose is below the lower limit of the target range (i.e., <110 mg/dl)	Reduce basal insulin dose by approximately 20%
Pre-lunch, pre-dinner, or bedtime blood glucose is below the lower limit of the target range (i.e., <110 mg/dl) despite the patient having consumed most of his/her meals	Reduce mealtime insulin by 20% at breakfast, lunch, or dinner, respectively
Steroid dose is being reduced	Decrease daily insulin dose by 20% for the next hospital day

Note that insulin may be initiated in insulin-naïve patients when they are admitted to the hospital at a total daily dose of 0.4 to 0.5 units per kg. Half the dose is given as basal insulin and, if patients are eating, the other half is given as mealtime insulin divided over the three meals (15).

Treating daytime hyperglycemia with basal insulin will increase the risk for nighttime or early morning hypoglycemia. In one recent study (14), most hospital hypoglycemia occurred in the early morning, which suggests the basal insulin dose was too high and perhaps the providers were reluctant to use fast-acting insulin analogs during the day.

The fast-acting analogs (lispro, aspart, or glulisine) may be administered with meals or after meals. Regular human insulin should not be used because the onset of action is about 1 hour after administration, and administering regular human insulin 30 to 60 min before the scheduled meal is not prudent due to unpredictability of the hospital setting, with late meal tray delivery or transport to radiologic procedures that may lead to delays in eating.

Despite the best efforts of medical providers, hypoglycemia may still occur. All units of the hospital should have nurses trained to recognize the signs and symptoms of hypoglycemia. Nurses should be trained on the use of hypoglycemia treatment protocols, which include the use of intravenous dextrose and glucagon injections. Furthermore, overtreatment of hypoglycemia may result in hyperglycemia and should be avoided.

Let us consider my patient, Mrs. K. The only insulin ordered was glargine, with the dose increased when the patient's blood sugars were high. There was no programmed mealtime insulin. The patient was improving and, after the amputation, the stress response waned and the basal insulin requirement decreased, but the dose of glargine was not adjusted downward. In addition, the patient was more sensitive to insulin due to chronic kidney disease. This patient was at risk for hypoglycemia from a standard insulin sliding scale, in which the same graded doses of insulin is given to correct for hyperglycemia in all patients.

I recently asked a colleague of mine why he thought the promise of intensive glycemic control did not pan out in recent clinical trials of intensive insulin therapy. He likened insulin to a Ferrari and we physicians are unskilled drivers. If we jump into the powerful car, we can reach our destination, unless, of course, we crash along the way. It is prudent then, to slow down, examine our hospital processes, and develop protocols and procedures that reduce the risk of hypoglycemia in our hospitalized patients.
